# DRDT: Distributed and Reliable Data Transmission with Cooperative Nodes for Lossy Wireless Sensor Networks

**DOI:** 10.3390/s100402793

**Published:** 2010-03-29

**Authors:** Jaewan Seo, Moonseong Kim, In Hur, Wook Choi, Hyunseung Choo

**Affiliations:** 1 Digital Media & Communication Business, Samsung Electronics, Korea; E-Mail: jaewan.seo@samsung.com; 2 Information and Communications Examination Bureau, Korean Intellectual Property Office, Korea; E-Mail: moonseong@kipo.go.kr; 3 Mobile Communications Company, LG Electronics, Korea; E-Mail: in.hur@lge.com; 4 Department of Computer Science and Engineering, Hankuk University of Foreign Studies, Korea; E-Mail: twchoi@hufs.ac.kr; 5 School of Information and Communication Engineering, Sungkyunkwan University, 440-746, Korea

**Keywords:** wireless sensor networks, reliable data transmission, modular approach, energy-efficiency, cooperative operation

## Abstract

Recent studies have shown that in realistic wireless sensor network environments links are extremely unreliable. To recover from corrupted packets, most routing schemes with an assumption of ideal radio environments use a retransmission mechanism, which may cause unnecessary retransmissions. Therefore, guaranteeing energy-efficient reliable data transmission is a fundamental routing issue in wireless sensor networks. However, it is not encouraged to propose a new reliable routing scheme in the sense that every existing routing scheme cannot be replaced with the new one. This paper proposes a *Distributed and Reliable Data Transmission (DRDT)* scheme with a goal to efficiently guarantee reliable data transmission. In particular, this is based on a pluggable modular approach so that it can be extended to existing routing schemes. DRDT offers reliable data transmission using neighbor nodes, *i.e.*, helper nodes. A helper node is selected among the neighbor nodes of the receiver node which overhear the data packet in a distributed manner. DRDT effectively reduces the number of retransmissions by delegating the retransmission task from the sender node to the helper node that has higher link quality to the receiver node when the data packet reception fails due to the low link quality between the sender and the receiver nodes. Comprehensive simulation results show that DRDT improves end-to-end transmission cost by up to about 45% and reduces its delay by about 40% compared to existing schemes.

## 1. Introduction

Guaranteeing energy-efficient reliable data transmission is a fundamental routing issue in wireless sensor networks. Many studies have assumed an ideal link model that guarantees successful transmission within the radio range [1,2]. However, recent studies have shown that under realistic circumstances links are highly unreliable due to various factors, such as interference, attenuation, and fading [3,4]. Particularly, wireless sensor networks have a higher packet loss ratio than other wireless network environments. Even though a retransmission mechanism is commonly used to recover from corrupted packets, this basic mechanism may considerably increase the number of retransmissions [5]. Therefore, it is crucial to consider having an energy-efficient reliable data transmission scheme.

Several routing schemes have been proposed to provide reliable data transmission for unreliable wireless links. Reliable Multi-Segment Transport for directed diffusion (RMST) [6] uses NACK model to guarantee both reliability and the order of fragment arrivals in Directed Diffusion [7]. Woo *et al*. [8] select reliable routes based on statistical link connectivity obtained from an EMWA estimator. Couto *et al*. [9] propose expected transmission count metric (ETX) to select routes to guarantee high data transmission rate and throughput. PRR×Distance greedy forwarding provides high energy-efficiency and reliability by a tradeoff between packet reception rate (PRR) and distance improvement to destination [10,11]. The authors of [12] propose Dynamic Switch-based Forwarding (DSF) that considers PRR and the low duty cycle networks when it selects the route to reduce sleep latency.

Basically, it is not encouraged to propose a new routing scheme for reliable data transmission in the circumstance where there are various routing schemes already implemented in sensor network applications. The reason is that it is practically not feasible to replace every routing scheme with the new one. Instead, it is more effective to adopt a pluggable modular approach that can be easily applied to an existing routing scheme. However, several issues should be considered for the modular approach to become a generalized solution to provide the reliability of all existing routing schemes. First, only a small amount of additional cost should be incurred in extending the existing routing schemes due to resource constraints such as energy, memory, and computing power. Second, the modular approach should not participate in path selection. This is performed in the network layer and reliability should be offered independently in a lower layer. Third, the modular approach should effectively reduce the number of retransmissions to achieve high energy-efficiency in data transmission.

In this paper, we propose a *Distributed and Reliable Data Transmission (DRDT)* scheme with a goal to efficiently provide reliable data transmission based on the modular approach. DRDT determines whether or not a neighbor node overhearing data packets transmitted to a receiver node will perform retransmission task on behalf of a transmitting node. This node is called a *helper node*. If a receiver node is located in an unreliable transmission region from the sender and fails to receive a data packet, the selected helper node retransmits the data packet. This enhances reliability of data transmission. In this case, the link quality of the helper node to the receiver node is superior to that of the sender node to the receiver node. Therefore, DRDT is able to reduce the number of retransmissions and guarantee energy-efficient data transmission. Our contributions are as follows:

- DRDT can be applied to all types of existing routing schemes since it operates as a pluggable extension layer under the network layer.
- DRDT effectively reduces the number of retransmissions by using helper nodes. The helper node selection is based on the link quality to the receiver node so that the retransmission of the helper node outperforms that of the sender node when the link quality between the sender and the receiver nodes is low.
- The helper node selection performs in a distributed manner. The sensor nodes which overhear the data packet decide whether to become helper nodes by themselves without exchanging any information with the neighbor nodes.
- DRDT guarantees high energy-efficiency and effectively reduces end-to-end transmission delay.

The remainder of this paper is organized as follows. In Section 2, we explain several schemes that provide reliable data transmission. We describe the proposed scheme in Section 3. The simulation results are presented in Section 4. Finally, Section 5 concludes this paper.

## 2. Related Work

The original greedy forwarding, a well-known routing scheme in wireless sensor networks, transmits data repeatedly by selecting a neighbor node that is closest to the destination as the next forwarding node [13,14]. Since it selects the next forwarding node using only the location information of the neighbor node, the original greedy forwarding is simple to realize and results in less load in selecting a neighbor node and in packet transmission. The original greedy forwarding also assumes an ideal environment that offers successful data transmission within a radio range. In realistic wireless sensor networks where the the quality of a link is unstable, link quality degrades as the link becomes distant. The original greedy forwarding selects a farthest neighbor node as the next forwarding node. This causes numerous retransmissions.

Several researches have pointed out how ideal radio models lead to wrong results in realistic wireless sensor networks. Ganesan *et al*. presented empirical results illustrating that packet loss can occur over a short distance since the distribution of packet reception over distance is not uniform [3]. The authors also proved that low-power environments quite commonly have asymmetric links. Kotz *et al*. [15] enumerated the set of common assumptions used in MANET research. They prove that these assumptions are usually incorrect. In unreliable links, the ideal connectivity graph can be a loss in terms of fading and obstacles. Zhao *et al*. [4] reported measurements of packet transmission in three different environments: an indoor office building, a habitat with moderate foliage, and an open parking lot. This research showed the spatiotemporal characteristics of packet loss and its environmental dependence. The results obtained from the SCALE connectivity assessment tool [16] demonstrated that packet reception rate can be changed irrespective of distance in an area of more than 50% of the radio range, where SCALE was used in [17] to develop statistical models based on link characteristics of wireless sensor networks.

A new greedy forwarding scheme that transmits data packet effectively to the destination in a realistic wireless sensor network has recently been proposed to prevent the selection of an unreliable link. Couto *et al*. [9] have measurements for DSDV and DSR over a 29-node 802.11b testbed and show that the minimum hop-count metric performs poorly since it does not take the channel characteristics into account, especially with the fact that minimizing the hop count maximizes the distance traveled by each hop and it is likely to increase the loss ratio. They presented the *expected transmission count metric* (ETX) that finds high-throughput paths by incorporating the effects of link loss ratio, asymmetry and interference. The authors of [18] compared the ETX metric to per-hop Round Trip Time (RTT) and per-hop packet pair metrics. This research found that the ETX metric has the best performance when all nodes are stationary.

Seada *et al*. propose the PRR×Distance Greedy Forwarding that selects a next hop by considering multiplication of PRR and the distance to the destination [10,11]. They proved, based on a mathematical analysis, that the energy efficiency is maximized when the data packet is transmitted to the node that has the largest value after the multiplication of the PRR by the distance improvement. Therefore, PRR × Distance Greedy Forwarding transmits data packets by multiplying the PRR between the sender node and the neighbor node and the distance improvement between the neighbor node and the destination, and selecting a neighbor node that has the largest value as the next forwarding node. That is, by selecting a forwarding node with a good PRR that is the link quality between the two nodes and high proximity to the destination. This scheme offers reliable transmission capability in an unstable link region that can cause packet loss.

The authors of [19] proposed Cluster-Based Forwarding (CBF) which can be expanded into the existing routing schemes as an extension layer. CBF guarantees reliable transmission using a cluster that consists of helper nodes with a good link quality among the neighbor nodes of the receiver node. The helper nodes, which substitute for the receiver node that fails to receive the data packet, cooperatively retransmit the data packet they overheard. However, selecting helper nodes requires that all nodes should know in advance the whole path information of the next forwarding node for the destination sink. Furthermore, if the location of the sink changes all the nodes should go through the process of selecting helper nodes again. This results in a high cost. Therefore, CBF cannot be the general solution to provide reliability to realistic wireless sensor networks.

## 3. Proposed Scheme

### 3.1. Link Loss Model

Analyzing and simulating a reliable data transmission scheme requires a realistic link loss model. In this paper, we use the PRR as the link quality between two nodes. The PRR ranges from 0 to 1 and is calculated using the following link loss model [20,21]:

(1) $$\mathrm{PRR}(d)={\left(1-\frac{1}{2}e^{-\frac{\gamma (d)}{2}\frac{1}{0.64}}\right)}^{16f-8l},$$

where, *d* is the distance between two nodes, *γ*(*d*) is a *signal to noise ratio* (SNR) for *d*, *f* is the length of a frame, and *l* is the length of a preamble. This model considers various radio parameters, such as a *path-loss exponent* (*η*) and a *log-normal shadowing variable* (*σ*). In the simulated environment, *η* and *σ* are 3.0 and 3.8, respectively. Equation (1) reflects MICA2 motes that use the NCFSK modulation method and Manchester Encoding. Other detailed information follows the MICA2 specifications [22].

**Figure 1.**
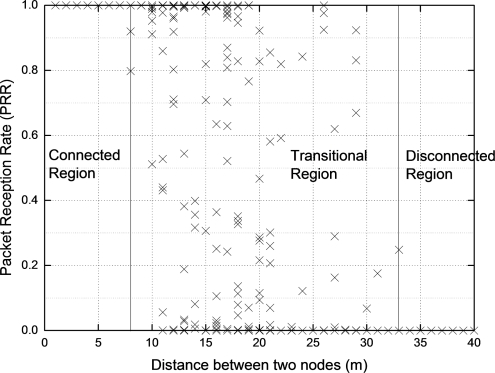
Packet reception rate of considered link loss model at different distance.

Recent researches [3,4,8,20,21] have shown three distinct data reception regions: connected, transitional, and disconnected. In the connected region, nodes can transmit packets reliably in the absence of congestion since PRR is always 1. In the transitional region, some links have high PRR, while others have the opposite. Therefore, when we design a routing scheme, we should consider this region. In the disconnection region, no links or very weak links exist. Figure 1 depicts the link layer model, where the observed connected region is from 0 to 8 *m*, the transition region is from 8 to 33 *m*, and the disconnected region is from 33 *m*.

### 3.2. Scheme Overview and Assumptions

**Figure 2.**
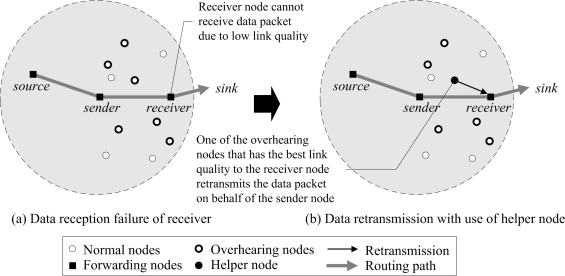
Overview of proposed DRDT scheme.

The proposed DRDT scheme guarantees reliable energy-efficient data transmission *via* the extension layer that operates under the network layer. The basic idea behind the proposed scheme is the cooperative retransmission of neighbor nodes that overhear the data packet transmitted to the receiver node, so as to increase the data transmission success rate and reduce the number of retransmissions. In Figure 2, every neighbor node of a receiver that overheard the data packet becomes a candidate helper node. Each decides if it should promote itself as a helper node using waiting time in a distributed manner. At this point, the helper node is selected by considering PRR to the receiver node, so that the number of retransmissions can be reduced. The selected helper node retransmits the data packet on behalf of the sender node when the receiver node failed to receive the data packet due to low PRR (Figure 2b). The helper node can effectively reduce the number of retransmissions since it uses a better PRR link than the sender node.

DRDT functions based on the following basic and general wireless sensor network environments.

- The network has relatively low congestion.
- Fixed sensor nodes with constant radio ranges are randomly distributed.
- Each sensor node knows itself and its geographical location information *via* GPS or the distribution-based localization algorithm.
- Each node knows the location information of the neighbor nodes and the PRR value between itself and the neighbor nodes by transmitting a ”hello” packet at regular intervals.
- The network uses a timeout-based ACK model to offer reliability.
- It is assumed that insignificant computing power is required for mathematical operations.

### 3.3. Distributed and Reliable Data Transmission: DRDT

The proposed scheme DRDT consists of two major phases: helper node selection phase using helper value and transmission phase. Performing the two phases repetitively, DRDT increases the reliability of the data transmission in an energy-efficient way. We first propose the helper value, followed by the helper node selection phase using the helper value.

#### Helper Value (*H*)

**Figure 3.**
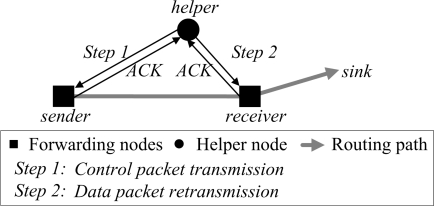
Retransmission of the helper node.

A helper node, which can retransmit data packet with low cost, should be selected among neighbor nodes that overhear the data packet sent to the receiver node in order for DRDT to provide reliability in an energy-efficient way. The expected cost is defined as the total transmitted bits until the receiver node obtains the data packet [19]. We first explain the procedure how the helper node retransmits the data packet when the receiver node fails to receive the data packet to calculate the expected cost (Figure 3).

Step 1: Helper node transmits a control packet to prevent unnecessary retransmissions of the sender.
Step 2: Helper node retransmits the overheard data packet to the receiver node.

For the receiver node to obtain the data packet successfully, the expected number of data packet transmissions is $\frac{1}{{\mathrm{PRR}}_{h\to r}\times {\mathrm{PRR}}_{r\to h}}$, where *h* is the helper and *r* is the receiver in these two steps. Similarly, the expected number of control packet transmissions is $\frac{1}{{\mathrm{PRR}}_{h\to s}\times {\mathrm{PRR}}_{s\to h}}$, where *s* is the sender. We assume that the data packet size ratio of a control packet to a data packet is *λ* to calculate the expected cost. Therefore, the expected cost to perform retransmission using a helper node can be written as follows.

(2) $$\mathrm{Expected}\;\mathrm{Cost}=\frac{1}{{\mathrm{PRR}}_{h\to r}\times {\mathrm{PRR}}_{r\to h}}+\frac{\lambda }{{\mathrm{PRR}}_{h\to s}\times {\mathrm{PRR}}_{s\to h}}$$

As we can see in Equation (2), minimizing the cost required to use a helper node on behalf of the sender node demands the maximization of the PRR between a helper node and the sender node, as well as the PRR between the helper node and the receiver node. That is, in an attempt to maximize the energy-efficient retransmission using a helper node, a node with a high PRR to both the receiver and the sender nodes is selected as a helper node.

The *helper value* (*H*) is the criterion to calculate the condition of such a helper node. The helper value is defined as follows:

(3) $$\begin{array}{l} H=w_{1}H_{\mathrm{PRR}}(s,c)+w_{2}H_{\mathrm{PRR}}(r,c),\\ w_{2}\geq w_{1}\geq 0\;\mathrm{and}\;w_{1}+w_{2}=1, \end{array}$$

where *c* denotes a current node that is considered as a helper node. *w*_1_ and *w*_2_ are the weighting values. Generally, the data packet size is larger than the control packet size, and therefore, a larger weighting value is applied to the PRR of the receiver node than to that of the sender node. *H_PRR_*(*a*, *b*) refers to the PRR between nodes *a* and *b*. It is defined as follows:

(4) $$H_{\mathrm{PRR}}(a,b)=1-\min(1,-{\text{log}}_{10}{\mathrm{PRR}}_{a\to b}\times {\mathrm{PRR}}_{b\to a})$$

In *H_PRR_* (*a*, *b*), logarithm function rapidly decreases the selected probability when PRR approaches low. In [10] and [11], when the number of retransmissions exceeds 10, the transmission is assumed to have failed. Therefore, if the PRR is below 0.1, the helper node cannot retransmit the data packet. Thus, this paper assumes the base of the log to be 10; this value can be changed.

#### Helper Node Selection Phase

DRDT causes low overhead because every node can decide to be a helper node in a distributed manner. A *waiting time* (*W*) is used to determine whether or not a node performs retransmission as a helper node. Each node that overhears the data packet transmitted to the receiver node first checks the packet header to determine if the destination is one of its neighbor nodes. If it is, the node waits for the waiting time with respect to its helper value. The waiting time is calculated as follows [23].

(5) W=(1−H)×δ,

where *δ* is the predefined maximum waiting time. According to Equation (5), the waiting time is obtained shorter, as the helper value increases higher. That is, the closer the helper value is to 1, the higher the probability the node will be selected as a helper node. Once the waiting time expires, a node selects itself as a helper node and broadcasts a request to send (RTS) packet to retransmit the data packet to the receiver node. If the other nodes receive the RTS packet from another node or overhear the data packet being transmitted to the receiver node while the waiting time has not expired, the nodes stop waiting (because they believe that another helper node with a better condition has been selected) and are removed from the helper node selection phase, and the data packets are dropped.

**Figure 4.**
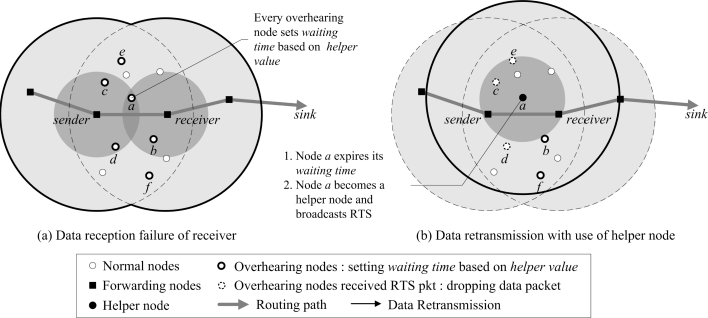
Helper node selection.

Figure 4 depicts the helper node selection phase based on a helper value. The nodes *a*, *b*, *c*, *d*, *e*, and *f* that overhear the data packet transmitted to the receiver node first calculate the helper values. They set the waiting time with respect to their helper values. In Figure 4a, the helper value of node *a* is 1 since it is located in the connected region where both the sender and receiver nodes overlap. While other nodes *b*, *c*, *d*, *e*, and *f* can all have helper values of 1 or lower, it is assumed for now that they all have helper values below 1. Therefore, node *a* sets the shortest waiting time by using Equation (5). Having set up the waiting time, node *a* selects itself as a helper node and makes other nodes know this by broadcasting an RTS packet. Here, the nodes that receive the RTS packet before their waiting time to be expired completely discard the data packet, as in nodes *c*, *d*, and *e* of Figure 4b.

If more than one helper nodes are selected in the helper node selection phase, duplicated retransmission is performed. There are two possible scenarios. First, nodes may not receive the RTS packet from selected helper node due to low link quality, as in nodes *b* and *f* of Figure 4b. In this case, they keep listening to the channel and still wait for their waiting time. Eventually, several helper nodes can be selected. This problem is easily solved by the transmission phase in the next subsection. The second scenario is that if a current node overhears the data packet transmitted from the helper node, it possibly starts the helper node selection phase again for the same data packet. This causes selecting unnecessary helper nodes over and over. To solve this, the helper node adds a *flag* bit set to 1 in the data packet before retransmission to differentiate the data packet from the sender node. By doing so, nodes do not start the helper node selection phase for the data packet from the helper node. Table 1 shows the pseudo-code of the helper node selection phase.

#### Transmission Phase

A helper node selected transmits an RTS packet once to determine if the receiver node needs retransmissions of the data packet and prevent other nodes from selecting themselves as helper nodes. If the receiver node that has not received the data packet, receives the RTS packet of the helper node, it transmits the clear to send (CTS) packet to the helper node. After receiving the CTS packet, the helper node begins its transmission phase. When it receives several RTS packets, the receiver node only executes the transmission phase for the first RTS packet, and ignores the others, in order to solve the duplicated retransmission problem by several helper nodes. The transmission phase is generally divided into two steps: the control packet transmission step which prevents the retransmission of the sender node and the data packet retransmission step which retransmits the data packet to the receiver node. In the control packet transmission step, the helper node sends out the control packet to the sender node to prevent data packet duplication due to retransmissions of the sender node. The helper node considers the PRR of the sender node, and the number of control packet retransmissions is effectively reduced. This step should be started before the data packet retransmission step since the sender node possibly retransmits the data packet during retransmission of the helper node. In the data packet retransmission step, the helper node on behalf of the sender node that has a low PRR value, retransmits the data packet to the receiver node. At this time the *flag* bit set to 1 is added in the data packet. Consequently, the helper node reduces the number of retransmissions by delegating the retransmission task from the sender node.

**Figure 5.**
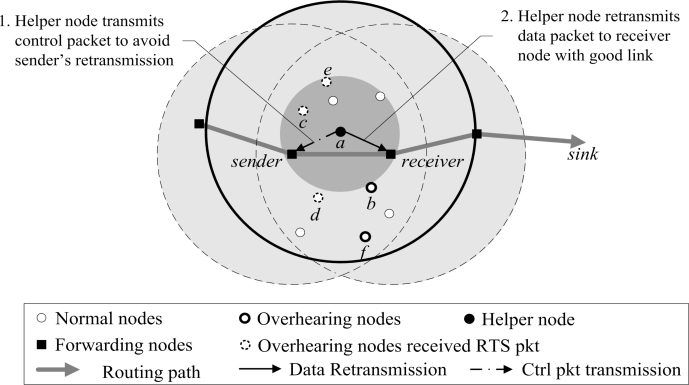
Transmission phase by helper node.

Figure 5 shows the data packet retransmission and the control packet transmission by helper node *a*. First, after receiving the CTS packet from the receiver node, helper node *a* retransmits the data packet to the receiver node. Helper node *a* also transmits the control packet to the sender node and the sender node transmits an ACK packet to the helper node, after receiving the control packet. In the process of transmitting the control packet, if the sender node overhears the data packet transmitted from the helper node, the sender node can transmit an ACK packet in advance, before receiving the control packet. This can prevent unnecessary energy consumption due to the control packet transmission. Table 2 shows the pseudo-code of the transmission phase.

## 4. Performance Evaluation

We implemented DRDT using C++ to evaluate its performance. Table 3 lists the main parameters. The radio model that reflects MICA2 motes is implemented with several adjustable parameters. 2,500 sensor nodes with 40 m radio range are randomly distributed in a network over an area of 300 *m* × 300 *m*. A sink is positioned at the center of the field, and each node transmits a data packet to the sink over multiple hops, using different routing protocols. We use Original Greedy Forwarding (termed GF-HOP) [13] and PRR × Distance Greedy Forwarding (termed GF-ETX) [11] as baseline routing schemes. DRDT is implemented as extension layer for each baseline routing scheme. We compare these schemes before and after applying CBF [19] and DRDT.

### 4.1. Number of Helper Nodes Used at Different PRR

**Figure 6.**
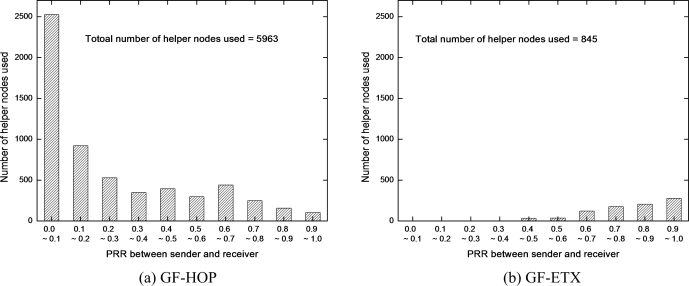
Number of helper nodes used at different PRR.

In this subsection, we show simulation results of the number of helper nodes at different PRR between sender and receiver nodes, when every node transmits data packets to the sink. That is, we correlate the number of helper nodes used of one node to its next hop PRR. We observe that most of the helper nodes belong to those nodes with weak links (PRR 0 ∼ 0.1) as shown in Figure 6a. That is, DRDT enhances reliability by cooperative retransmissions of helper nodes over links with low PRR. In Figure 6b, however, most of the helper nodes perform the retransmission task over links with good PRR since GF-ETX has already selected fairly good paths through built-in PRR based measures. In this case, helper nodes on good links can still work when the backward PRR of the receiver node is very low. This also helps enhance GF-ETX performance.

### 4.2. End-to-End Transmission Cost

End-to-end transmission cost is defined as the ratio of total number of packets in bits sent by all nodes to the total number of useful packets received by the sink. This can be written as [24]

(6) $$\mathrm{Transmission}\;\;\mathrm{Cost}=\frac{T}{U\cdot N}$$

where *N* represents the average number of hops in paths, *T* shows the total bits each node transmits, and *U* denotes the number of useful bits that the sink received. Equation (6) reflects both communication overhead and energy-efficiency.

**Figure 7.**
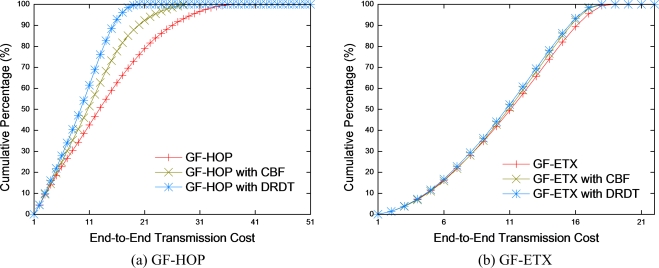
End-to-end transmission cost.

GF-HOP transmits the data packet to the next hop node that is closest to the sink without consideration of PRR. Consequently, PRR between two nodes is not sound. This leads to high transmission cost, as the number of retransmissions increases radically, as shown in Figure 7a. To reduce the transmission cost, retransmission tasks of the helper node is very important. In GF-HOP with CBF, the number of retransmissions considerably diminishes, as it receives the data packet through helper nodes that have good PRR to the receiver node. However, CBF has problems stemming from inefficient use of control packets, such as RTS/CTS/ACK since CBF only considers PRR of the sender when selecting the helper node. GF-HOP with DRDT has a relatively high energy-efficiency compared to GF-HOP with CBF. DRDT effectively transmits the data and control packets since the helper node is selected by considering the PRR of both the receiver and the sender nodes.

There is not much difference between GF-ETX with CBF and GF-ETX with DRDT as shown in Figure 7b because GF-ETX finds a next hop with the value of the product of PRR and distance. Therefore, the performance of GF-ETX with CBF and that of GF-ETX with DRDT are restricted by transmitting the data packet through the better route with good PRR. However, in GF-ETX with DRDT, performance is enhanced by helper nodes in some parts with poor PRR region.

### 4.3. End-to-End Transmission Delay

**Figure 8.**
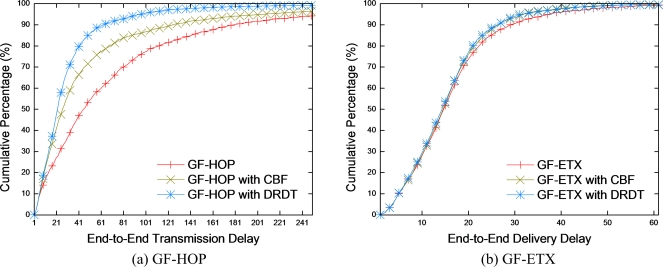
End-to-end transmission delay.

End-to-end transmission delay represents the unit time that the data packet takes from source to sink. If the ACK model does not receive the ACK packet by the timeout, after transmitting the data packet, it provides reliability using a retransmission mechanism. GF-HOP in Figure 8a attributes the lengthened delay to the timeout resulting from numerous retransmissions. Conversely, in GF-HOP with DRDT, the delay decreases due to the reduction of the number of retransmissions. The longer the timeout setting, the bigger the difference between performance of GF-HOP with DRDT and that of the former schemes would be made. In the case of GF-ETX, in Figure 8b, it is noticeable that there is a decline of delay due to the reduction in the number of retransmissions. However, in certain network regions (e.g., a network boundary) every node can be in a transmission region with low PRR. Therefore, it is identified that GF-HOP and GF-ETX may both experience noticeably prolonged delay.

### 4.4. End-to-End Transmission Rate

In this subsection, we vary node density from 25 to 200 (*nodes/range*) and show its effect on the transmission rate. We use networks of 2,500 nodes and set the number of ARQ retransmission to 10, since ARQ with a limited number of retransmissions is normally the practical choice for implementation. Transmission rate is the percentage of data packets sent by the source node and received by the destination.

**Figure 9.**
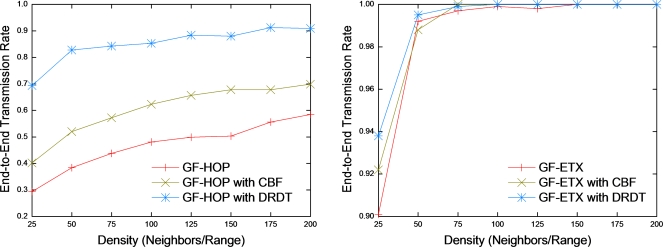
End-to-end transmission rate.

In general, the transmission rate of GF-HOP is low (Figure 9a). We observe that the transmission rates of GF-HOP with CBF and GF-HOP with DRDT are also low at density 25 due to the local minimum problem [25,26]. However, in the case of GF-HOP with CBF and GF-HOP with DRDT, the transmission rate increases more as the density increases compared to GF-HOP. Especially, GF-HOP with DRDT has the best performance compared to GF-HOP with CBF because DRDT uses helper nodes more effectively than CBF does as the same reason that we explain in subsection 4.2. As shown in Figure 9b, the transmission rate of GF-ETX is above 90%. However, it does not guarantee a 100% transmission rate until the density is 150, since the number of retransmissions exceeds 10 over links with low PRR. This is due to transmission failures that could still occur in terms of greedy disconnections. We observe a slight performance improvement brought about by DRDT in this case.

## 5. Conclusions

Energy-efficient reliable transmission is a fundamental routing issue in lossy wireless sensor networks. We have presented detail study about existing routing schemes to guarantee reliability in an efficient way. However, these reliable routing schemes only work well for specific applications which means that they cannot be used as a general solution to provide reliability. To solve the problem, we have proposed a DRDT scheme. DRDT enhances reliability in transmission by cooperative retransmission task of helper nodes. Furthermore, DRDT is extendable, as well as adaptable to existing routing schemes since DRDT operates as an extension layer under the network layer. Therefore, we believe that DRDT based on a pluggable modular approach can be a general solution in providing reliability to the existing routing schemes. Simulation results, in which GF-HOP is implanted as a basic routing scheme, indicate that poor performance of GH-HOP can be improved by applying DRDT. DRDT enhances end-to-end transmission cost and reduces its delay as a result of high transmission rate. In case of GF-ETX after applying DRDT, slight improvement is observed over these performance metrics. This means that DRDT performs retransmission task with very low overhead. Furthermore, DRDT demonstrates superiority in data retransmission compared to CBF.

In our upcoming research, we plan to study the scheduling of sensor motes to extend DRDT as a more practical scheme. We expect that consideration of low duty cycle networks helps to prolong operational time. However, end-to-end transmission cannot afford to maintain an always-awake transmission which leads a decrease the number of overhearing nodes. The performance metrics such as end-to-end transmission cost and delay can be reduced since improvements of DRDT depend on the number of overhearing nodes. Therefore, we also plan to analyze the interaction between low duty cycle networks and the number of overhearing nodes. The interesting overhearing phenomenon is expected according to duty-cycle networks.

## Figures and Tables

**Table 1.** Pseudo-code of helper node selection phase.

Input: sender id (*s_id*), receiver id (*r_id*), PRR between current and sender nodes (*PRR_c→s_*, *PRR_s→c_*), PRR between current and receiver nodes (*PRR_c→r_*, *PRR_r→c_*), *flag* bit (flag)
Output: helper node id (*h_id*)
HELPER NODE SELECTION (*s_id*, *r_id*, *PRR_c→s_*, *PRR_s→c_*, *PRR_c→r_*, *PRR_r→c_*, *flag*)
// When a current node overhears the data packet it starts helper node selection phase
1 *H*, *W*, *h_id* ← 0
2 **if***r_id* = one of neighbor nodes id of current node **and** flag ! = 0
// If *flag* value is 1, it means that the data packet is transmitted from helper node
3 **then***H_PRR_*(*c*, *s*) ← 1 − *min*(1, − log_10_*PRR_c→s_* × *PRR_s→c_*)
4 *H_PRR_*(*c*, *r*) ← 1 − *min*(1, − log_10_*PRR_c→r_* × *PRR_r→c_*)
5 *H* ← *w*_1_*H_PRR_*(*c*, *s*) + *w*_2_*H_PRR_*(*c*, *r*)
// Helper value calculation
6 **else** drop the data packet
7 **exit**
8 **if***H*! = 0
9 **then***W* ← (1 − *H*) × *δ*
// Set waiting time based on helper value
10 TIMER(*W*)
// Start timer with waiting time
11 **else** drop the data packet
12 **exit**
13 **while***W* > 0
14 **do if** overhearing the data packet again **or** overhearing the RTS packet
15 **then** drop the data packet
16 **exit**
17 *h_id* ← current node id
18 BROADCAST(*h_id*)
// Current node becomes the helper node and broadcasts RTS packet
// once with its id to neighbor node

**Table 2.** Pseudo-code of transmission phase.

Input: sender id (*s_id*), receiver id (*r_id*)
Output: retransmission task on behalf of sender node
TRANSMISSION PHASE (*s_id*, *r_id*)
// When a node is selected as a helper node, it starts transmission phase
1 *flag* ← 0
2 **if** receiving CTS packet from receiver node
3 **then** TRANSMIT(control, *s_id*)
4 TRANSMIT(data, *r_id*)
5 **else** drop the data packet
TRANSMIT (*packet_type, node_id*)
1 **if***packet_type* = *data*
2 **then***flag* ← 1
// If *flag* value is 1, it means that the data packet is transmitted
// from helper node add flag bit to packet header
3 transmit packet to *node_id* until receiving ACK packet
4 **return** 0

**Table 3.** Simulation parameters.

**Radio**			
Modulation	NCFSK	Encoding	Manchester
Output Power	−5 *dBm*	Frame Size	50 *Bytes*
**Transmission Medium**			
Path Loss Exponent	3	*PLD*_0_	55 *dBm*
Noise Floor	−105 *dBm*	*D*_0_	1 *m*
**Deployment Configuration**			
Area Height	300 *m*	Area Width	300 *m*
Number of Nodes	2,500	Radio Range	25 *m*
**Baseline Routing Schemes**			
Original Greedy Forwarding (termed GF-HOP) PRR×Distance Greedy Forwarding (termed GF-ETX)			
**Performance Metrics**			
End-to-End Transmission Cost / End-to-End Transmission Delay / End-to-End Transmission Rate			
